# Hepatic Steatosis and Cardiovascular Risk in Obesity: A 10‐Year Follow‐Up Study From Childhood Into Young Adulthood

**DOI:** 10.1111/ijpo.70148

**Published:** 2026-09-02

**Authors:** Anne‐Sophie R. Stroes, D. Meeike Kusters, Laura Draijer, Anneloes E. Bohte, Aart J. Nederveen, Eric de Groot, Marc A. Benninga, Adriaan G. Holleboom, Bart G. P. Koot, Barbara A. Hutten

**Affiliations:** ^1^ Department of Pediatric Gastroenterology Emma Children's Hospital, Amsterdam University Medical Centers, University of Amsterdam Amsterdam the Netherlands; ^2^ Amsterdam Gastroenterology Endocrinology Metabolism Research Institute Amsterdam the Netherlands; ^3^ Amsterdam Reproduction and Development Research Institute Amsterdam the Netherlands; ^4^ Department of Vascular Medicine Amsterdam University Medical Centers, University of Amsterdam Amsterdam the Netherlands; ^5^ Amsterdam Cardiovascular Sciences Research Institute Amsterdam the Netherlands; ^6^ Department of Pediatrics Amsterdam University Medical Centers, University of Amsterdam Amsterdam the Netherlands; ^7^ Department of Epidemiology and Data Science Amsterdam University Medical Centers, University of Amsterdam Amsterdam the Netherlands; ^8^ Department of Radiology and Nuclear Medicine Amsterdam University Medical Centres Amsterdam the Netherlands; ^9^ Imagelabonline and Cardiovascular Diepenheim the Netherlands

**Keywords:** carotid intima‐media thickness, childhood obesity, metabolic dysfunction‐associated steatotic liver disease, non‐alcoholic fatty liver disease, subclinical atherosclerosis

## Abstract

**Background:**

The early vascular impact of metabolic dysfunction‐associated steatotic liver disease (MASLD) remains unclear. This study examined associations of steatosis in childhood and young adulthood with cardiovascular risk in adulthood.

**Methods:**

Children with severe obesity were investigated for MASLD and cardiovascular risk, with 10‐year follow‐up. Hepatic steatosis was measured using proton magnetic resonance spectroscopy and cardiovascular risk by ultrasound carotid intima‐media thickness (cIMT). Participants were categorised into four trajectory groups based on presence of steatosis in childhood and/or adulthood: ‘absent’, ‘diminishing’, ‘adult‐onset’ and ‘persistent’. Associations between steatosis (presence and change over time) and cIMT, and between childhood metabolic factors and cIMT progression, were evaluated.

**Results:**

Of 52 participants, 46% had steatosis in childhood (‘diminishing’ or ‘persistent’), and 46% in adulthood (‘adult‐onset’ or ‘persistent’). Childhood steatosis was not associated with cIMT in adulthood, whereas adulthood steatosis was independently associated with increased cIMT (mean difference 0.035 mm, 95% CI: 0.010–0.060 mm; *p*‐adjusted = 0.008). Mean cIMT at follow‐up increased progressively across steatosis trajectory groups; (adjusted *p*‐for‐trend = 0.003). Childhood steatosis was the only metabolic factor associated with cIMT progression (*β* = 0.006, *p* = 0.030).

**Conclusions:**

Hepatic steatosis in young adulthood is independently associated with increased cardiovascular risk, and childhood steatosis relates to cIMT progression, supporting early MASLD detection and prevention.

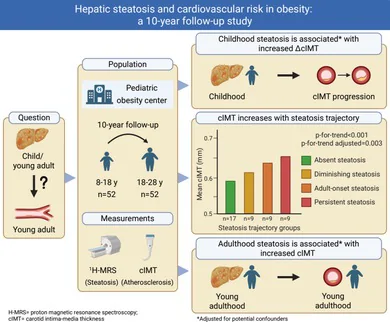

## Graphical abstract

Ten‐year follow‐up in obesity showing that childhood hepatic steatosis is associated with cIMT progression into young adulthood, cIMT in young adulthood increases stepwise according to steatosis trajectory, and young adulthood steatosis is independently associated with increased cIMT at that age.

**Abbreviations**
^1^H‐MRS **=**
: proton magnetic resonance spectroscopy
BMI: body mass index
cIMT: carotid intima‐media thickness
CVD: cardiovascular disease
HOMA‐IR: homeostatic model assessment of insulin resistance
LDL‐c: low‐density lipoprotein cholesterol
MASLD: metabolic dysfunction‐associated steatotic liver disease
NAFLD: non‐alcoholic fatty liver disease
SBP: systolic blood pressure
SD: standard deviation
TG: triglycerides

## 1 Introduction

Childhood obesity is a well‐known risk factor for cardiovascular disease (CVD), with longitudinal studies demonstrating a two‐ to threefold increase in CVD mortality amongst individuals with obesity during childhood and adolescence [1, 2]. This is particularly concerning given that the prevalence of childhood obesity has tripled over the past three decades, affecting 6.8% of children and adolescents worldwide in 2021 [3].

The increased cardiovascular risk associated with childhood obesity is partly attributable to coexisting cardiometabolic risk factors, such as dyslipidaemia, elevated blood pressure, hyperglycaemia and insulin resistance [4, 5]. However, childhood obesity is also involved in a direct atherogenic process. Pathological and epidemiological studies have shown that childhood obesity is independently associated with preclinical atherosclerosis in adulthood, as measured by carotid intima‐media thickness (cIMT) [6, 7]. These direct effects are largely mediated by chronic low‐grade inflammation and oxidative stress, which promote a proatherogenic state [8, 9, 10].

Metabolic dysfunction‐associated steatotic liver disease (MASLD), defined as hepatic steatosis in the presence of overweight or obesity or another cardiometabolic risk factor [11, 12], is highly prevalent amongst children with obesity and is increasingly recognised as an important contributor to cardiovascular risk [13, 14, 15]. As with obesity, the cardiovascular risk associated with MASLD is partly mediated by coexisting metabolic disturbances [16], but MASLD is also involved in a direct atherogenic process primarily via disturbed lipid metabolism and inflammatory cytokines [17, 18, 19, 20].

Although childhood obesity has been linked to increased cIMT [21], the long‐term impact of paediatric MASLD on cardiovascular risk remains unclear. Previous studies have reported inconsistent associations between hepatic steatosis and cIMT in childhood [22, 23], whilst most cross‐sectional adult studies do demonstrate a positive association between MASLD and cIMT [24]. This raises questions about the long‐term impact of early‐life steatosis on the trajectory of atherosclerotic cardiovascular risk into young adulthood.

In the present study, we therefore aimed to investigate whether the presence of hepatic steatosis at childhood age or young adult age is associated with cIMT measured at young adult age. Our secondary aims were [1] to examine how cIMT varies across different trajectories of steatosis, and [2] to identify whether childhood metabolic factors are associated with cIMT progression.

## 2 Methods

### 2.1 Study Design and Population

This prospective cohort study included children and adolescents who were evaluated for MASLD, formerly non‐alcoholic fatty liver disease (NAFLD), before inclusion in a lifestyle intervention programme at a specialised obesity centre in the Netherlands between 2008 and 2010 [25]. At baseline, participants underwent proton magnetic resonance spectroscopy (^1^H‐MRS) to quantify hepatic steatosis, and a subgroup also underwent ultrasound to measure cIMT [22]. Inclusion criteria were age between 8 and 18 years, primary obesity, and a body mass index (BMI) equivalent to an adult index of 35 kg/m^2^. Exclusion criteria were co‐existing liver disease or diabetes, current or previous use of steatogenic or oral antidiabetic medication, alcohol intake of ≥ 7 U/week or contraindications for MR scanning. All 119 original participants were invited for follow‐up 10 years later [26]. The study protocol was approved by the Medical Ethics Committee of Amsterdam University Medical Centres (NL66187.018.18). Written informed consent was obtained from all participants or their caregivers at both baseline and follow‐up.

### 2.2 Clinical Assessment

Anthropometric measurements, including height, weight and waist circumference, were performed at both baseline and follow‐up. Blood pressure was measured according to standardised protocols. Fasting blood samples were collected to assess glucose metabolism, lipid profiles and liver enzymes [26].

### 2.3 Steatosis Measurement

Hepatic steatosis was quantified using ^1^H‐MRS performed on the same 3.0‐T MR system device (Philips Healthcare, Best, the Netherlands) at both baseline and 10‐year follow‐up, as previously described [22, 26]. A liver fat fraction of > 1.8% on ^1^H‐MRS was used to define steatosis, a threshold validated for this device to correspond to > 5% steatosis on histology [27]. MRI technicians were blinded to clinical data.

### 2.4 cIMT Measurement

cIMT was measured at baseline and 10‐year follow‐up. High‐resolution B‐mode ultrasound images of the common carotid artery, carotid bulb, and internal carotid artery segments were assessed bilaterally, following a standardised protocol [22]. An Acuson Aspen instrument (Siemens Medical Solutions, Erlangen, Germany) equipped with a linear‐array transducer (L7, 5–12 MHz) was used. Images were analysed off‐line by an image analyst. The sonographer and analyst were blinded to clinical information. The far wall intima‐media thickness measurements of the six carotid segments were averaged to a mean cIMT per participant [22].

### 2.5 Statistical Analysis

Descriptive statistics were used to summarise demographic and clinical characteristics of the study population. Differences in continuous variables between groups were evaluated using independent‐samples *t*‐tests (for normally distributed data) or Mann–Whitney tests (for skewed data); chi‐squared tests were applied to compare categorical variables.

Associations between steatosis (at baseline or follow‐up) and cIMT at follow‐up were examined using linear regression analysis. Multivariable models were applied to adjust for potential confounders. These confounders were selected based on clinical relevance and the univariable associations between patient characteristics and either the determinant (presence of steatosis) or the outcome (cIMT at follow‐up). All potential confounders were included in the linear model (full model) and with stepwise backward elimination (*p* > 0.1 for removal) a final model, which always contained the determinant (presence of steatosis), was derived.

Participants were categorised into four steatosis trajectory groups: ‘absent steatosis’ (no steatosis at both timepoints), ‘diminishing steatosis’ (steatosis in childhood only), ‘adult‐onset steatosis’ (steatosis in adulthood only) and ‘persistent steatosis’ (steatosis at both timepoints). Differences in cIMT across groups were compared using linear regression models.

Associations of childhood cardiometabolic parameters (steatosis, BMI *Z*‐score, systolic blood pressure [SBP], triglycerides [TG], low‐density lipoprotein cholesterol [LDL‐c] and homeostatic model assessment of insulin resistance [HOMA‐IR]) with cIMT progression were similarly examined using linear regression with stepwise backward elimination. cIMT progression was calculated as the difference between cIMT at follow‐up and at baseline, divided by the difference in age at follow‐up and at baseline.

All *p*‐values are two‐sided, and *p*‐values < 0.05 were considered statistically significant. Analyses were performed using SPSS software version 24.0 (SPSS Inc., Chicago, IL).

## 3 Results

### 3.1 Description of the Study Population

The study population comprised 52 of the 119 participants (44%) from the original cohort, with a mean duration of follow‐up of 10.3 years (standard deviation [SD] 1.3). Fifteen individuals could not be reached due to invalid contact details, and 52 declined participation, most commonly due to lack of interest or time. Demographic and clinical characteristics of the study cohort at baseline and follow‐up are shown in Table 1. The mean age at baseline was 14.1 years (SD 2.3), and the mean age at follow‐up was 24.3 years (SD 2.8). The mean BMI at baseline was 38.5 kg/m^2^ (SD 5.4), and the mean BMI at follow‐up was 40.3 kg/m^2^ (SD 7.0). Eighteen participants (35%) were male. Three participants, all with MASLD at baseline, developed Type 2 diabetes mellitus and 70% had dyslipidaemia at follow‐up. During the follow‐up period, 28 (55%) received lifestyle interventions guided by a health professional and 16 participants (31%) underwent bariatric surgery. At follow‐up, 59% of participants reported that they were physically active, averaging 3 h/week.

**TABLE 1:** Patient characteristics of the study population at baseline and 10‐year follow‐up.

	At baseline, *N* = 52	At follow‐up, *N* = 52
Male sex—no. (%)	18 (34.6)	18 (34.6)
Age (years)—mean (SD)	14.1 (2.3)	24.3 (2.9)
Body mass index (kg/m^2^)—mean (SD)	38.5 (5.4)	40.3 (7.0)
Body mass index *Z*‐score—mean (SD)	4.1 (0.4)	3.9 (0.9)
Waist (cm)—mean (SD)	104 (10)	115 (18)
Systolic blood pressure (mmHg)—mean (SD)	123 (15)	121 (14)
Diastolic blood pressure (mmHg)—mean (SD)	81 (10)	74 (11)
Smoking—no. (%)	2 (5.0)	20 (38.5)
Ethnicity—no. (%)		
Dutch	37 (71.2)	37 (71.2)
Moroccan	1 (1.9)	1 (1.9)
Surinamese—East Indian	1 (1.9)	1 (1.9)
Turkish	7 (13.5)	7 (13.5)
Other	6 (10.3)	6 (10.3)
Biochemical/laboratory		
ALT (IU/L)—median (IQR)	27 (17–43)	21 (15–30)
AST (IU/L)—median (IQR)	25 (21–32)	21 (18–26)
γGT (IU/L)—median (IQR)	19 (17–28)	19 (13–37)
Total cholesterol (mmol/L)—mean (SD)	4.0 (0.7)	4.4 (0.7)
HDL cholesterol (mmol/L)—mean (SD)	1.1 (0.3)	1.3 (0.4)
LDL cholesterol (mmol/L)—mean (SD)	2.5 (0.6)	2.6 (0.7)
Triglycerides (mmol/L)—median (IQR)	0.8 (0.6–1.2)	0.8 (0.6–1.1)
Glucose (mmol/L)—mean (SD)	4.9 (0.5)	5.2 (1.7)
Insulin (μU/mL)—median (IQR)	103 (64–168)	64 (40–97)
HOMA‐IR—median (IQR)	3.0 (2.0–5.8)	2.2 (1.2–3.2)
Steatosis—no. (%)	24 (46)	24 (46)
Bariatric surgery—no. (%)	—	16 (31)
Lifestyle interventions—no. (%)	—	28 (55)

Abbreviations: ALT, alaninaminotransferase; AST, aspartaataminotransferase; HDL, high‐density lipoprotein; IQR, interquartile range; LDL, low‐density lipoprotein; No., number; SD, standard deviation; γGT, gamma‐glutamyltransferase.

### 3.2 Steatosis and cIMT Measurements

Of all participants, 17 (33%) had no steatosis at either timepoint (‘absent steatosis’), 9 (17%) had steatosis in childhood which diminished by adulthood (‘diminishing steatosis’), 9 (17%) had steatosis only in adulthood (‘adult‐onset steatosis’) and 15 (29%) had steatosis in both childhood and adulthood (‘persistent steatosis’). All those with steatosis at any time‐point met the diagnostic criteria for MASLD [11, 12]. Mean cIMT at baseline (*n* = 40) was 0.476 mm (SD 0.075), and mean cIMT at follow‐up (*n* = 52) was 0.620 mm (SD 0.049).

### 3.3 Association Between Childhood Steatosis and cIMT in Young Adulthood

In childhood (baseline), 24 participants (46%) had steatosis. Characteristics of participants with and without steatosis in childhood are summarised in Table S1. Mean cIMT at follow‐up was 0.638 mm (SD 0.048) for participants with childhood steatosis and 0.604 mm (SD 0.045) for those without childhood steatosis (mean difference: 0.034 mm, 95% CI 0.008–0.060 mm; *p* = 0.012; Table 2). After adjustment for potential confounders, this difference was slightly reduced and no longer statistically significant (final model: mean difference 0.019 mm, 95% CI −0.005 to 0.044 mm; *p* = 0.116; Table 2).

**TABLE 2:** Association between presence of steatosis in childhood and carotid intima‐media thickness in young adulthood.

	Unadjusted		Adjusted (full model)^a^		Adjusted (final model)^b^	
	*β* (95% CI)	*p*	*β* (95% CI)	*p*	*β* (95% CI)	*p*
Steatosis (yes/no)	0.034 (0.008–0.060)	0.012	0.025 (−0.002 to 0.052)	0.067	0.019 (−0.005 to 0.044)	0.116

Abbreviation: CI, confidence interval.

^a^ Adjusted for sex, body mass index, systolic blood pressure, triglycerides and HOMA‐IR.

^b^ Adjusted for sex, systolic blood pressure.

### 3.4 Association Between Young Adulthood Steatosis and cIMT in Young Adulthood

In young adulthood (10‐year follow‐up), 24 participants (46%) had steatosis, of which 17 participants already had steatosis in childhood. Characteristics of participants with and without steatosis in adulthood are shown in Table S2. Mean cIMT at follow‐up was 0.647 mm (SD 0.037) for participants with steatosis in adulthood and 0.599 mm (SD 0.045) for those without steatosis in adulthood (mean difference: 0.048 mm, 95% CI 0.024–0.071 mm; *p* < 0.001). After adjustment for potential confounders, cIMT remained significantly higher in participants with steatosis in adulthood compared with those without (final model: mean difference 0.035 mm, 95% CI 0.010–0.060 mm, *p* = 0.008, Table 3).

**TABLE 3:** Association between presence of steatosis and carotid intima‐media thickness in young adulthood.

	Unadjusted		Adjusted (full model)^a^		Adjusted (final model)^b^	
	*β* (95% CI)	*p*	*β* (95% CI)	*p*	*β* (95% CI)	*p*
Steatosis (yes/no)	0.048 (0.024–0.071)	< 0.001	0.023 (−0.010 to 0.056)	0.161	0.035 (0.010–0.060)	0.008

Abbreviation: CI, confidence interval.

^a^ Adjusted for sex, body mass index, systolic blood pressure, triglycerides and HOMA‐IR.

^b^ Adjusted for sex.

### 3.5 Comparison of cIMT in Young Adulthood Between Steatosis Trajectory Groups

**FIGURE 1:**
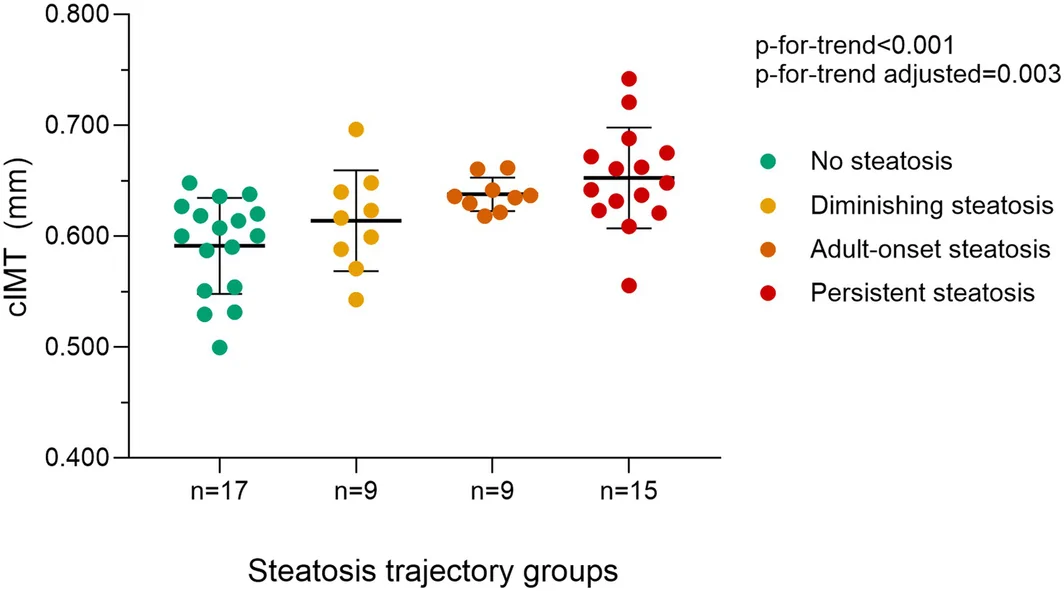
Individual cIMT values according to steatosis groups. Individual values are shown, with group mean ± SD indicated. Steatosis groups: ‘absent steatosis’ = no steatosis in either childhood or adulthood, ‘diminishing steatosis’ = only steatosis at childhood age, ‘adult‐onset steatosis’ = only steatosis at young adult age, ‘persistent steatosis’ = steatosis at both childhood and young adult age. *p*‐for‐trend adjusted: Adjusted for sex. cIMT, carotid intima‐media thickness.

Figure 1 shows the mean cIMT at follow‐up for participants with ‘absent steatosis’, ‘diminishing steatosis’, ‘adult‐onset steatosis’ and ‘persistent steatosis’ as 0.591 mm (SD 0.043), 0.614 mm (SD 0.045), 0.638 mm (SD 0.015) and 0.653 mm (SD 0.045), respectively (*p*‐for‐trend < 0.001; *p*‐for‐trend adjusted for sex = 0.003). Mean cIMT was lower in the ‘absent steatosis’ group compared with the ‘persistent steatosis’ and ‘adult‐onset steatosis’ group (*p*‐adjusted = 0.008 and *p*‐adjusted = 0.014, respectively; Table S3). A marked difference between these groups was the proportion of bariatric surgery; this was highest in the ‘diminishing steatosis’ group and lowest in the ‘adult‐onset’ group (56% vs. 11%, *p* = 0.046).

### 3.6 Metabolic Determinants of cIMT Progression

Of the clinically relevant childhood metabolic factors examined, childhood steatosis was the only significant metabolic determinant of cIMT progression from childhood to young adulthood across multiple regression models (*β* = 0.006, *p* = 0.030). None of the other metabolic factors were independently associated with cIMT progression, including serum lipids despite their established association with atherosclerosis (Table S4).

## 4 Discussion

This 10‐year prospective cohort study of children and young adults with obesity demonstrates that the presence of hepatic steatosis in childhood was not significantly associated with cIMT in young adulthood, whereas steatosis in young adulthood was independently associated with increased cIMT at that age. In addition, cIMT in young adulthood increased stepwise according to steatosis trajectory: it was lowest in the absence of steatosis, higher in childhood‐only steatosis, further increased in adult‐onset steatosis and highest in persistent steatosis. Childhood steatosis was the only metabolic determinant associated with cIMT progression from childhood to adulthood, indicating that early‐life steatosis contributes to the gradual development of subclinical atherosclerosis over time. Taken together, these findings strongly suggest that the vascular impact of MASLD becomes apparent already in young adulthood.

There is inconsistent evidence on the association between steatosis and cIMT in childhood. Several paediatric MASLD studies that relied on ultrasound for steatosis assessment reported higher cIMT already in childhood [23, 28], whilst studies using liver biopsy or MRS did not find such an association [25, 29]. The association between childhood steatosis and cIMT progression into adulthood observed in our cohort extends these findings, suggesting that vascular alterations associated with steatosis may become more apparent during the transition from childhood to young adulthood.

In young adulthood, the presence of steatosis was independently associated with increased cIMT. These findings are in line with studies in late adulthood showing an association of MASLD with increased cIMT independent of metabolic syndrome [24], whereas our study extends these findings into young adulthood.

Longitudinal observation in our cohort showed a stepwise increase in cIMT according to steatosis trajectory (progressing from absent, to diminishing, to adult‐onset and to persistent steatosis). This pattern suggests that improvement in hepatic steatosis at an early age may mitigate cardiovascular risk [13, 30], which is a relevant finding for clinical practice. Collectively, our results emphasise not only the importance of early intervention in childhood, but also the potential effectiveness of interventions in reducing long‐term cardiovascular risk.

Bariatric surgery was performed in 31% of participants in young adulthood and may have contributed to resolution of steatosis, as bariatric surgery was more common in the diminishing steatosis group (56%). In adult studies, bariatric surgery appears effective in reducing atherosclerosis [31], although its use in children and adolescents is still debated and evolving with the emergence of pharmacological options [32, 33, 34]. GLP‐1 receptor agonists were not widely available yet during the study period and were not used by any of the participants, nor did participants use other weight‐loss medications.

The increased cIMT associated with hepatic steatosis may be partly attributable to coexisting cardiometabolic risk factors, for which we adjusted our models. The atherosclerotic process may be further enhanced by atherogenic lipid abnormalities hallmarking MASLD, as well as by insulin resistance driven by increased free fatty acid accumulation [16]. However, neither serum lipids nor glycaemic parameters were associated with cIMT progression in our cohort. Beyond these indirect effects, MASLD may contribute further to atherosclerosis via chronic low‐grade inflammation and endothelial dysfunction, leading to a proatherogenic state [17, 18, 19]. Recent experimental evidence also suggests that hepatic steatosis can directly promote atherosclerosis progression through the release of small extracellular vesicles from steatotic hepatocytes, which impair cholesterol efflux in macrophages and stimulate foam cell formation [35].

Strengths of this study include its longitudinal design, which allowed assessment of both timing and progression of the effect of steatosis on early cardiovascular risk. Additionally, steatosis was assessed using MRS, which is highly accurate. MRS and cIMT measurements were performed by a blinded single operator, minimising intraobserver variability, precluding interobserver variability and reducing the risk of bias. Several limitations of our study should be noted. First, the relatively small sample size at follow‐up may have limited the statistical power to detect smaller true effects, and could have introduced bias due to selective loss to follow‐up. The follow‐up rate in this study was relatively low, which is inherent to a long‐term follow‐up study. However, there were no significant differences in baseline characteristics between the complete baseline cohort and the smaller follow‐up cohort [26]. Given the small sample size, the subgroup analysis in which the cohort was divided into four groups warrants careful interpretation. Detailed information on long‐term adherence to lifestyle changes during the 10‐year follow‐up period was not available, which limits our ability to assess their contribution to steatosis trajectories. Despite including all major cardiometabolic risk factors in the analyses, the observed association between steatosis and cIMT could be influenced by other confounding factors. Despite these methodological limitations, the consistency of our findings in both the subgroup analysis and the overall association analyses supports the robustness of our results.

## 5 Conclusion

In conclusion, our findings show that hepatic steatosis at young adult age is independently associated with increased cIMT, and childhood steatosis is associated with cIMT progression into adulthood. cIMT also increases stepwise according to steatosis trajectory (from absent steatosis, to diminishing steatosis, to adult‐onset steatosis and to persistent steatosis). Together, these results demonstrate that the vascular impact of MASLD begins early in life and accumulates over time. These findings underscore the importance of early detection and management of MASLD in children to prevent long‐term cardiovascular morbidity.

## Author Contributions

D.M.K., L.D., B.G.P.K. and B.A.H. created the study design; D.M.K., L.D., E.G., B.G.P.K. and B.A.H. collected the data; A.‐S.R.S. and B.A.H. analysed the data; A.‐S.R.S., D.M.K., B.G.P.K. and B.A.H. interpreted the data; A.‐S.R.S. wrote the manuscript draft and D.M.K., B.G.P.K. and B.A.H. were involved in writing of the manuscript. All authors critically revised the manuscript and gave final approval of the submitted and published versions.

## Funding

This study was funded by Vaillant fonds. The funding source was not involved in the collection, analysis and interpretation of data; nor in the writing of the report; and had no involvement in the decision to submit the article for publication.

## Conflicts of Interest

A.G.H. has been a consultant for Novo Nordisk, Inventiva and Echosens. B.G.P.K. has been a consultant for Boehringer Ingelheim and Inventiva. B.A.H. has received consultant fees and research grants from Silence Therapeutics, all of which were paid to her institution, as well as travel and accommodation support from Ultragenyx. The other authors declare no conflicts of interest.

## Supporting information


**Table S1:** Patient characteristics for children with and without steatosis at baseline.
**Table S2:** Patient characteristics for young adults with and without steatosis at follow‐up.
**Table S3:** Differences in mean cIMT between the steatosis groups.
**Table S4:** Association between childhood cardiometabolic factors and cIMT progression into young adult age.

## Data Availability

The data that support the findings of this study are available from the corresponding author upon reasonable request.
