# Supplementary material for: Hepatic Steatosis and Cardiovascular Risk in Obesity: A 10‐Year Follow‐Up Study From Childhood Into Young Adulthood
Source: Pediatr Obes. 2026 Sep 2;21(9):e70148. doi: 10.1111/ijpo.70148 (PMC13536924; doi:10.1111/ijpo.70148)
Supplement: Supplementary file 1 [file IJPO-21-e70148-s001.pdf]

SUPPLEMENTARY MATERIAL

Hepatic Steatosis and Cardiovascular Risk in Obesity:

a 10-Year Follow-up Study from Childhood into Young Adulthood

Anne-Sophie R. Stroes[1,2,3], D. Meeike Kusters[4,5,6,7], Laura Draijer[1], Anneloes E. Bohte[8], Aart J. Nederveen[8], Eric de Groot[7,9], Marc A. Benninga[1,2,3], Adriaan G. Holleboom[2,4], Bart G.P. Koot[1,2,3], Barbara A. Hutten[2,5,7].

[1] Department of Pediatric Gastroenterology, Emma Children's Hospital, Amsterdam University Medical Centers, location University of Amsterdam, Amsterdam, The Netherlands.

[2] Amsterdam Gastroenterology Endocrinology Metabolism research institute, Amsterdam, The Netherlands.

[3] Amsterdam Reproduction and Development Research Institute, Amsterdam, The Netherlands.

[4] Department of Vascular Medicine, Amsterdam University Medical Centers, location University of Amsterdam, Amsterdam, The Netherlands.

[5] Amsterdam Cardiovascular Sciences research institute, Amsterdam, the Netherlands

[6] Department of Pediatrics, Amsterdam University Medical Centers, location University of Amsterdam, Amsterdam, The Netherlands.

[7] Department of Epidemiology and Data Science, Amsterdam University Medical Centers, location University of Amsterdam, Amsterdam, The Netherlands.

[8] Department of Radiology and Nuclear Medicine, Amsterdam University Medical Centres, Amsterdam, The Netherlands.

[9] Imagelabonline and Cardiovascular, Diepenheim, the Netherlands.

Corresponding author:

Anne-Sophie R. Stroes, MD

Department of Pediatric Gastroenterology and Hepatology

Emma Children's Hospital, Amsterdam University Medical Centres

Meibergdreef 9, 1005 AZ Amsterdam, The Netherlands

Email: a.r.stroes@amsterdamumc.nl

Telephone: + 31 20 566 9111

Supplementary Table S1. Patient characteristics for children with and without steatosis at baseline

	Steatosis n=24	No steatosis n=28	p-value
Male sex – no. (%)	12 (50.0)	6 (22.2)	0.038
Age (years) – mean (SD)	14.3 (2.2)	13.8 (2.3)	0.442
Body mass index (kg/m^2) – mean (SD)	40.0 (4.8)	37.3 (5.7)	0.097
BMI Z-score	4.2 (0.4)	3.9 (0.5)	0.038
Waist (cm) – mean (SD)	108 (9)	100 (10)	0.006
Systolic blood pressure (mmHg) – mean (SD)	127 (14)	119 (16)	0.079
Diastolic blood pressure (mmHg) – mean (SD)	84 (12)	77 (8)	0.023
Smoking – no. (%)	1 (5%)	1 (5%)	0.970
Biochemical/Laboratory			
ALT (IU/L) – median (IQR)	36 (21-49)	27 (16-36)	0.116
AST (IU/L) – median (IQR)	27 (22-40)	24 (20-31)	0.178
yGT (IU/L) – median (IQR)	26 (18-36)	18 (16-23)	0.007
Total cholesterol (mmol/L) – mean (SD)	4.2 (0.7)	3.8 (0.6)	0.057
HDL cholesterol (mmol/L) – mean (SD)	1.1 (0.3)	1.0 (0.2)	0.361
LDL cholesterol (mmol/L) – mean (SD)	2.6 (0.6)	2.4 (0.6)	0.302
Triglycerides (mmol/L) – median (IQR)	0.9 (0.7-1.4)	0.8 (0.5-1.1)	0.023
Glucose (mmol/L) – mean (SD)	5.1 (0.5)	4.6 (0.4)	<0.001
Insulin (µU/mL) – median (IQR)	152 (80-188)	82 (53-110)	0.002
HOMA-IR – median (IQR)	4.6 (2.6-6.7)	2.3 (1.4-3.5)	0.003

Abbreviations: ALT, alanine aminotransferase; AST, aspartate aminotransferase; yGT, gamma-glutamyltransferase; HDL, high-density lipoprotein; IQR, interquartile range; LDL, low-density lipoprotein; mmol/L, millimoles per litre; No, number; SD, standard deviation.

Supplementary Table S2. Patient characteristics of young adults with and without steatosis at follow-up

	Steatosis n=24	No steatosis n=28	p-value
Male sex – no. (%)	14 (58)	4 (16)	0.002
Age (years) – mean (SD)	25 (2.9)	24 (3.0)	0.442
Body mass index (kg/m^2) – mean (SD)	43.5 (6.7)	37.1 (6.3)	0.001
BMI Z-score	4.3 (0.6)	3.5 (0.9)	0.002
Waist (cm) – mean (SD)	128 (13)	103 (11)	<0.001
Systolic blood pressure (mmHg) – mean (SD)	129 (14)	114 (10)	<0.001
Diastolic blood pressure (mmHg) – mean (SD)	77 (12)	72 (9)	0.065
Smoking – no. (%)	12 (50)	8 (31)	0.166
Biochemical/Laboratory			
ALT (IU/L) – median (IQR)	29 (21-68)	17 (13-21)	<0.001
AST (IU/L) – median (IQR)	25 (21-33)	19 (1.5-21.5)	0.003
γGT (IU/L) – median (IQR)	37 (22-66)	14.0 (10.0-19.5)	<0.001
Total cholesterol (mmol/L) – mean (SD)	4.6 (0.7)	4.2 (0.7)	0.045
HDL cholesterol (mmol/L) – mean (SD)	1.2 (0.3)	1.4 [0.4]	0.029
LDL cholesterol (mmol/L) – mean (SD)	2.8 (0.7)	2.5 (0.7)	0.202
Triglycerides (mmol/L) – median (IQR)	1.1 (0.8-1.6)	0.6 (0.5-0.8)	<0.001
Glucose mmol/L – mean (IQR)	5.7 (2.4)	4.7 (0.4)	0.054
Insulin (µU/mL) – median (IQR)	95 (64-133)	45 (26-72)	<0.001
HOMA-IR – median (IQR)	3.0 (2.2-4.9)	1.4 (0.8-2.5)	<0.001
Childhood steatosis	15 (63)	9 (33)	0.049
Bariatric surgery	5 (21)	11 (42)	0.104

Abbreviations: ALT, alanine aminotransferase; AST, aspartate aminotransferase; γGT, gamma-glutamyltransferase; HDL, high-density lipoprotein; IQR, interquartile range; LDL, low-density lipoprotein; mmol/L, millimoles per litre; No, number; SD, standard deviation.

Supplementary Table S3. Differences in mean cIMT between the steatosis groups

	cIMT difference (mm) mean (95% CI)	p-value
Persistent steatosis vs. no steatosis	0.061 (0.032 to 0.090)	<0.001^
	0.032 (-0.008 to 0.071)	0.117*
	0.044 (0.012 to 0.076)	0.008#
Persistent steatosis vs. diminishing steatosis	0.039 (0.004 to 0.073)	0.030^
	-0.006 (-0.055 to 0.042)	0.791*
	0.019 (-0.018 to 0.057)	0.305#
Persistent steatosis vs. adult-onset steatosis	0.015 (-0.020 to 0.049)	0.399^
	-0.001 (-0.040 to 0.039)	0.972*
	0.002 (-0.033 to 0.037)	0.898#
Adult-onset steatosis vs. no steatosis	0.047 (0.013 to 0.081)	0.008^
	0.032 (-0.005 to 0.070)	0.090*
	0.042 (0.009 to 0.074)	0.014#
Adult-onset steatosis vs. diminishing steatosis	0.024 (-0.015 to 0.063)	0.222^
	-0.006 (-0.052 to 0.040)	0.803*
	0.017 (-0.021 to 0.055)	0.368#
Diminishing steatosis vs. no steatosis	0.023 (-0.011 to 0.057)	0.188^
	0.038 (0.001 to 0.075)	0.047*
	0.025 (-0.008 to 0.057)	0.135#

^Unadjusted
*Full model: Adjusted for sex, body mass index, systolic blood pressure, triglycerides, and HOMA-IR
#Final model: Adjusted for sex
Abbreviations: cIMT, carotid intima-media thickness; CI, confidence interval; HOMA-IR, homeostatic model assessment of insulin resistance; vs, versus.

Supplementary Table S4. Association between childhood cardiometabolic factors and cIMT progression into young adult age

	Beta (se)	p-value
Childhood steatosis (yes versus no)	0.006 (0.019)	0.030
Childhood systolic blood pressure	<0.0001 (<0.0001)	0.064
Childhood triglycerides	0.003 (0.002)	0.174
Childhood BMI Z-score	-0.007 (0.004)	0.059

Abbreviation: BMI, body mass index.
